# Psilocybin: From Serendipity to Credibility?

**DOI:** 10.3389/fpsyt.2021.659044

**Published:** 2021-04-21

**Authors:** James J. Rucker, Allan H. Young

**Affiliations:** ^1^Department of Psychological Medicine, King's College London, Institute of Psychiatry, Psychology, and Neuroscience, London, United Kingdom; ^2^South London and Maudsley National Health Service Foundation Trust, Bethlem Royal Hospital, Beckenham, United Kingdom

**Keywords:** psilocybin, clinical trials, retreat center, depression, therapy

## Abstract

Psilocybin has a long history of non-medical use and some seem to infer from this that it has therapeutic utility. Early phase clinical trials with psilocybin are encouraging, but suggest only that larger, multicentre trials are required. These are ongoing but will take many years to complete. Meanwhile, retreat centers offering paid experiences with psilocybin truffles have opened in some countries, often using early phase clinical trial data as a basis for bold, public facing claims. This seems unwise. Early phase trials are not designed for their results to be generalized outside the setting they were undertaken in. To do so risks being misleading. Providing what may be seen as an unregulated drug intervention as a paid service is difficult to reconcile with long-held ethical principles underpinning human research and treatment development that were laid down by the 1947 Nuremberg Code and the 1962 Kefauver Harris Amendments. By using psilocybin before it has been properly tested, retreat centers may be undermining their own credibility and the credibility of the wider field.

Drugs are molecular tools waiting for their use to be found. In psychiatry, this sometimes happens with a sprinkling of serendipity and, occasionally, self-experimentation.

That moment of serendipity for psilocybin, the active ingredient of so-called “magic” mushrooms, was probably many thousands of years ago. Evidence from antiquity suggests psilocybin mushrooms were used by different, geographically separated ancient cultures (1, 2), with the most prominent evidence from ancient meso-American cultures, who described psilocybin mushrooms as “teonanacatl” (“flesh of god”). Today, around 2.5 million individuals in the UK population report a lifetime history of use of psilocybin mushrooms, a proportion that has been stable over the last 10 years (3). A US population estimate suggested a slightly higher proportion (4).

However, to determine whether a drug should become a licensed medicine, an objective process of scientific enquiry is required. This usually consists of set of gold standard clinical trials, defined by international agreement (5). In contrast of the history of psilocybin use, clinical trials, in their modern and robust forms, have taken place only for the past 50 years or so.

This has introduced a problem. For some who are well-versed with the history and non-medical use of psilocybin, there is a strong tendency to infer that it has a confirmed therapeutic utility. This is reflected in a vocal minority who call for legitimization and liberalization of use. They have had some success, with changes to statutes in the US state of Oregon that challenge decades of prohibition and make non-medical use more likely. Whilst the justifications for unregulated psilocybin use are understandable, they are also non-objective, insofar as they infer a utility on the basis of historical precedent, anecdotal evidence and (sometimes) personal experience. In medicine, justification of use in these terms (particularly as a treatment for others) is viewed with strong suspicion.

There is reasoned foundation for such suspicion. History suggests that presumption of safety and efficacy of drugs without exhaustive scrutiny is dangerous. The current clinical trials process is based, amongst other things, on the Kefauver Harris Amendments of US statute in 1962 (6). These were a logical response to the tragedy that occurred with the drug thalidomide that, marketed in 1957 for morning sickness in pregnant women without adequate testing of safety, caused thousands of children to be born with life-altering birth abnormalities (7). The Kefauver Harris Amendments introduced a legal requirement for manufacturers of drugs for human use to provide proof of effectiveness and safety prior to marketing. They were an application of principles laid down by the Nuremberg Code of 1947 (8), which is a set of ethical statements about human research that arose from the Nuremberg trials at the end of the Second World War. The Declaration of Helsinki by the World Medical Association in 1964 further enshrined these principles in clinical trials (9). Taken together, they form the cornerstone of human research ethics and have guided regulations for the development of new treatments for human use ever since.

A natural extension of these principles is that it is unethical and potentially dangerous to infer safety and efficacy of a treatment before a burden of objective proof has been met. Here, the burden of proof is a series of gold-standard, randomized, controlled trials (RCTs) that collect evidence about a potential treatment's safety and effectiveness prior to it being considered for wider public use. Clinical trials must be undertaken according to strict guidelines that protect the rights of participants (5). For example, usually it would be unethical to require a participant to pay for their participation in a clinical trial.

Those clinical trials proceed in 4 “phases.” Phase 1 are first-in-human trials. These establish basic safety, usually in healthy volunteers who are paid for their participation. Phase 2 are first-in-patient trials. These establish feasibility of a new intervention in a patient population with a particular diagnosis. Phase 3 are efficacy trials. These are randomized, controlled trials, often in very large numbers of similar patients in numerous centers around the world. Phase 3 trials often cost hundreds of millions of dollars and take many years to complete. It is only phase 3 trials that are used to make licensing decisions, because only phase 3 trials have sufficiently robust designs to inform those decisions. Even after licensing, phase 4 trials investigate treatments further, often picking up rare side effects that phase 3 trials can't detect. Licenses are sometimes withdrawn on the basis of phase 4 trials. Even after this, drug safety monitoring is essentially endless, and drugs may be withdrawn for safety reasons after being on the market for many years. A good example of this is the antidepressant nefazodone.

A hypothesis currently is that psilocybin, given in a medically controlled setting along with psychological support, is a safe and effective treatment for major depressive disorder. A hypothesis makes no presumption about the truth, but it is the basis for a process of scientific enquiry that addresses the need for a burden of proof. Since psilocybin is a drug, this burden of proof is a set of gold standard clinical trials. To date, this burden of proof has not been met for psilocybin. Whilst we are making some progress, there is a long way still to go.

We completed a gold standard Phase 1 trial in 2019 at King's College London (10). This was the largest ever randomized controlled trial of psilocybin in healthy volunteers. 89 participants were randomized to receive a single dose of either placebo, 10 or 25 mg of psilocybin. We used a proprietary formulation of psilocybin, developed and manufactured by COMPASS Pathways PLC, who also funded the study. The objectives were to assess the short-term effects of psilocybin on emotional processing and cognitive function at 1 and 4 weeks, as measured by the CANTAB cognitive battery and a panel of social and emotional cognition scales. We also measured adverse event rates and serious adverse event rates between the three trial arms. Follow up was for 12 weeks.

The results from this study are currently under peer review, however we presented a synthesis of adverse event data in poster form recently (10). The results were reassuring. No clinically serious adverse events were recorded. No adverse event led to a participant withdrawing from the trial. Sixty-seven percent of all adverse events appeared and resolved on the day of dosing. Ninety-two percentage of adverse events likely to be “psychedelic” in nature were resolved by the next day. Those remaining were usually positive in nature. Altered mood was one of the most frequently recorded adverse events, however by *post-hoc* analysis 96% were judged to be either positive or neutral in nature. We concluded that psilocybin was well-tolerated and was not associated with significant negative effects on measures of cognitive and emotional processing in healthy volunteers.

If the results from this study are confirmed, this represents a significant step in the regulatory process that may (or may not) lead to licensing of psilocybin therapy. Randomized, single-center phase 2 trials with psilocybin in patients have also been completed, for example in major depressive disorder (11) and cancer related anxiety (12, 13). Randomized, single center trials that will report through 2021 and 2022 include participants with major tobacco addiction (NCT01943994) and obsessive-compulsive disorder (NCT03356483). Together, these make a convincing case for larger, more extensive trials in patients.

These are now underway. A multi-center RCT of psilocybin assisted therapy in 216 participants with treatment resistant depression (NCT03775200) started in 2019 in Europe and North America, funded by COMPASS (14). A multi-center RCT of psilocybin therapy in 80 participants with major depressive disorder (non-treatment resistant) (NCT03866174) started in 2019 in the United States, funded by the Usona Institute. Results from both of these trials are expected in 2022. Both of these trials are a credible basis for phase 3 trials, and phase 3 trials are used by regulators to make licensing decisions and issue marketing authorisations. It is legally incompatible for a drug with a marketing authorization to remain within Schedule 1. If psilocybin receives a marketing license then, similar to formulations of cannabis with approved medical use, it will likely be rescheduled. This will be a very significant step, challenging 50 years of prohibition.

However, whilst this is all underway (it takes many years) private retreat centers advertising packages that include dosing sessions with psilocybin have opened in countries such as the Netherlands. Here, more relaxed approaches to regulation allow psilocybin-containing truffles to be offered legally, outside the usual processes of drug regulation.

Websites of retreat centers promote narratives of personal growth, emotional breakthroughs and spiritual development. They use the results of early phase clinical trials with psilocybin to bolster claims to potential customers, despite the fact that those trials were never designed for that purpose and should not be extrapolated in this way. Some retreat centers publicly advertise the results of their own research programmes to potential customers, without pointing out that such research is likely to be biased. It is usual for packages that include a single psilocybin dosing session to cost many thousands of Euros.

Whilst retreat centers are not acting illegally and many clearly intend to provide high quality experiences to customers, there are several potential problems here. The problem of how to manage the clinical risks of customers who will bring whole lifetimes of psychology to a potentially life changing experience. Looked at another way, what is the center actually providing, if it is not a drug treatment? The problem of how to justify the use of an evidence base for the center when that evidence base was never meant for that. Moreover, how will they maintain quality and appropriate levels of care when there will be other centers attempting to undercut prices and there is no external process of regulation?

Underlying much of this is the fact that psilocybin is not certified as a treatment, or an intervention, for anything. Complicating the picture is that it is also legally defined in many countries as a drug with significant dangers. Whilst that definition is questionable, it is nonetheless the default position of people external to the psilocybin field. Whilst it is a “Devil's advocate” position, if psilocybin isn't a licensed treatment (but is being investigated as one) and the legal position is that it is a drug with significant dangers, then retreat centers logically run the risk of being seen by authorities as unregulated centers of human experimentation where participants are paying for their own dosing with a drug legally classified as potentially dangerous. It was just this sort of scenario that the Nuremberg Code and the Declaration of Helsinki were set up to address.

Yet, it gets more complicated still. Some governments have specifically issued exemptions from regulations to allow the use of drugs like psilocybin (although not specifically psilocybin) when they have an established role as a religious sacrament. For example, the US government allows the indigenous peoples of North and Central America to use mescaline-containing peyote cactus in a ceremonial setting. Governments in South America take a similar view of DMT-containing ayahuasca, long used ceremonially there.

Is it possible, therefore, to justify the existence of retreat centers on religious or spiritual grounds, thus bypassing the need for regulation? On balance, probably not. Religious use long predates scientific and medical trials of psilocybin. There is no reason why retreat centers could not have been set up prior to medical research, were they motivated by religious or spiritual use. The rapid development of retreat centers subsequent to medical research, does not suggest religious or spiritual motivations are a significant factor.

Is it possible for retreat centers to justify their existence on the basis of the prevalence of recreational use and a harm minimization argument? Since so many people are doing it anyway, why not provide a safe and supportive context? Again, likely not. There is no convincing, substantive evidence that providing psilocybin in a retreat center is more safe or more supportive than standard harm reduction advice. This is that recreational users take sensible precautions, such as using psilocybin mushrooms of known quality at home, with sober companions that they trust to take care of them and a pre-agreed process for contacting further help, if necessary.

Is it possible for retreat centers to justify their existence on the basis of existing trial evidence? The answer to this is definitely not. At the moment, the clinical evidence for psilocybin as a treatment is very limited. All clinical trials to date are pilot trials and feasibility RCTs taking place in single centers with carefully selected groups of patients and highly motivated teams of researchers (11–19). Whilst it is true that almost all report encouraging treatment effect sizes, it is usual for early phase clinical trials to over-estimate these. Currently, reported effect sizes for psilocybin treatment are far larger than is clinically credible, or likely in the “real world.” Beyond the basic problem that the effect sizes are likely to be over-estimates, single center trials say nothing about whether a treatment generalizes outside the center the trial was done. To test this, multi-center trials are needed. These have not yet been completed with psilocybin.

Why are we saying this? We lead a research team investigating psilocybin. We are “representatives of the establishment”: established medics with all the motivations to maintain the current hegemony and power structures that were associated with the prohibition of classical psychedelics in the first place. We have pointed out the problems of retreat centers with a business model in the same article that we describe our collaboration with a for-profit commercial company developing psilocybin through medical licensing. Moreover, one of the authors (JR) works for a (government regulated) medical cannabis clinic in the UK, prescribing cannabis products that have not been subject to the very process we are arguing psilocybin should be subject to. Surely, we are hypocrites? Surely, we are biased ourselves.

Indeed, we are. However, if we are then we are in a context where processes of external regulation provide a necessary balance to that. Clinical trials and the proper processes of regulation of human research were set up to guard against the worst excesses of quackery and hearsay that used to define medical treatment. They are based upon the core ethical principles surrounding human research that most people support. As such, they garner the broader trust of governments and wider society. We think that this trust is very important given the tentative position the field finds itself with psilocybin. If you don't trust us, then we'd like to appeal that you consider trusting an internationally agreed process forged from the lessons of history.

Our central point is that if psilocybin is ever to achieve wider credibility in society, then it is this regulated process of clinical trials that is most likely to achieve it. Few in society could object to a societal and legal reorientation of approach toward psilocybin if it was shown by proper clinical trial evidence to have a use in healthcare. But this hasn't happened yet. Offering psilocybin to people outside a regulated context, particularly in the context of payment, risks undermining the process of legitimately challenging psilocybin's legal status.

A current psilocybin retreat center says the following on its public facing website:

“All of this research, regardless of its focus, comes together to produce one clear message: psychedelics are powerful, and can transform your life. Indeed, it is this increasingly strong evidence-base for its benefits that has helped transform psilocybin from a banned substance to an FDA-approved medicine: trials which are set to render it available as a prescription-based panacea for treatment-resistant depression are currently underway in Europe and North America.” (20).

Psilocybin is not an “FDA-approved medicine.” No clinical trial can be “set to render” anything. Indeed, to presume this (and that it is a “panacea”) is the sort of quackery that clinical trials were set up to counter, and which we argue is liable to undermine legitimate attempts to investigate the safety and effectiveness of psilocybin.

History relates that the current landscape with psilocybin is familiar territory. Unevidenced, ideological eulogy along with widespread recreational self-experimentation with psychedelics (including psilocybin) in the 1960s led (in part) to the stringent, criminal legal restrictions in place in countries around the world. Fifty years later these laws are still in place, with all of the stigma they have created. We are faced with an uphill battle to persuade governments and the public that psilocybin may have a place in healthcare. Attempting to circumvent established processes of regulation by offering psilocybin outside of a regulated setting is liable to lead to a form of redress, just as it did before. In time, we hope that the clinical trial evidence, properly collected, may make an argument that few could ignore. Perhaps, until that time, we would be better to retain a collective position of clinical equipoise.

## Data Availability Statement

The original contributions generated in the study are included in the article/supplementary material, further inquiries can be directed to the corresponding author.

## Ethics Statement

The studies involving human participants were reviewed and approved by London—Brent Research Ethics Committee. The patients/participants provided their written informed consent to participate in this study.

## Author Contributions

JR wrote this manuscript. AY commented and made amendments to the manuscript. Both authors contributed to the article and approved the submitted version.

## Conflict of Interest

This work presents independent research part-funded by the National Institute for Health Research (NIHR) Biomedical Research Centre at South London and Maudsley NHS Foundation Trust and King's College London. JR is an honorary consultant psychiatrist at The South London & Maudsley NHS Foundation Trust, a consultant psychiatrist at Sapphire Medical Clinics and an NIHR Clinician Scientist Fellow at the Centre for Affective Disorders at King's College London. James Rucker's salary is funded by a fellowship (CS-2017-17-007) from the National Institute for Health Research (NIHR). JR leads the Psychedelic Trials Group with Professor Allan Young at King's College London. King's College London receives grant funding from COMPASS Pathways PLC to undertake phase 1 and phase 2 trials with psilocybin. COMPASS Pathways PLC has paid for JR to attend trial related meetings and conferences to present the results of research using psilocybin. JR asserts that COMPASS Pathways had no influence over the content of this article. JR has undertaken paid consultancy work for Beckley PsyTech and Clerkenwell Health. Payments for consultancy work are received and managed by King's College London. JR does not benefit personally. JR has no shareholdings in pharmaceutical companies. AY employed by King's College London; Honorary Consultant SLaM (NHS UK) Paid lectures and advisory boards for the following companies with drugs used in affective and related disorders: Astrazenaca, Eli Lilly, Lundbeck, Sunovion, Servier, Livanova, Janssen, Allegan, Bionomics, Sumitomo Dainippon Pharma, COMPASS. Consultant to Johnson & Johnson. Consultant to Livanova. AY received honoraria for attending advisory boards and presenting talks at meetings organised by LivaNova. Principal Investigator in the Restore-Life VNS registry study funded by LivaNova. Principal Investigator on ESKETINTRD3004: “An Open-label, Long-term, Safety and Efficacy Study of Intranasal Esketamine in Treatment-resistant Depression.” Principal Investigator on “The Effects of Psilocybin on Cognitive Function in Healthy Participants.” Principal Investigator on “The Safety and Efficacy of Psilocybin in Participants with Treatment-Resistant Depression (P-TRD).”
